# ECG Imaging to Detect the Site of Ventricular Ischemia Using Torso Electrodes: A Computational Study

**DOI:** 10.3389/fphys.2019.00050

**Published:** 2019-02-11

**Authors:** Vinay Kara, Haibo Ni, Erick Andres Perez Alday, Henggui Zhang

**Affiliations:** ^1^Biological Physics Group, School of Physics and Astronomy, The University of Manchester, Manchester, United Kingdom; ^2^Department of Pharmacology, The University of California, Davis, Davis, CA, United States; ^3^Division of Cardiovascular Medicine, Oregon Health and Science University, Portland, OR, United States; ^4^School of Computer Science and Technology, Harbin Institute of Technology, Harbin, China; ^5^China Space Institute of Southern China, Shenzhen, China

**Keywords:** ECGI, ventricle, torso electrodes, inverse problem, ventricular ischemia, regularisation methods

## Abstract

Electrocardiography provides some information useful for ischemic diagnosis. However, more recently there has been substantial growth in the area of ECG imaging, which by solving the inverse problem of electrocardiography aims to produce high-resolution mapping of the electrical and magnetic dynamics of the heart. Most inverse studies use the full resolution of the body surface potential (BSP) to reconstruct the epicardial potentials, however using a limited number of torso electrodes to interpolate the BSP is more clinically relevant and has an important effect on the reconstruction which must be quantified. A circular ischemic lesion on the right ventricle lateral wall 27 mm in radius is reconstructed using three Tikhonov methods along with 6 different electrode configurations ranging from 32 leads to 1,024 leads. The 2nd order Tikhonov solution performed the most accurately (~80% lesion identified) followed by the 1st (~50% lesion identified) and then the 0 order Tikhonov solution performed the worst with a maximum of ~30% lesion identified regardless of how many leads were used. With an increasing number of leads the solution produces less error, and the error becomes more localised around the lesion for all three regularisation methods. In noisy conditions, the relative performance gap of the 1st and 2nd order Tikhonov solutions was reduced, and determining an accurate regularisation parameter became relatively more difficult. Lesions located on the left ventricle walls were also able to be identified but comparatively to the right ventricle lateral wall performed marginally worse with lesions located on the interventricular septum being able to be indicated by the reconstructions but not successfully identified against the error. The quality of reconstruction was found to decrease as the lesion radius decreased, with a lesion radius of <20 mm becoming difficult to correctly identify against the error even when using >512 torso electrodes.

## Introduction

Cardiovascular disease is the most common cause of morbidity and mortality in developed countries. Although atrial arrhythmias are more common, ventricular arrhythmias are the more lethal arrhythmias accounting for around 50% of all sudden cardiac deaths (Huikuri et al., 2001). It is well known that myocardial ischemia predisposes to ventricular tachyarrhythmias and fibrillation (Ghuran and Camm, 2001), and early and effective diagnosis of myocardial ischemia is most essential for proper treatment of patients with cardiac ischemia in order to save their lives.

Electrocardiography (ECG) provides some information useful for ischemic diagnosis. Conventionally, a standard 12-lead ECG is widely used to detect abnormalities in the heart electrical activity, therefore, provides a convenient way for detection of myocardial ischemia (Sejersten et al., 2007). However, due to a low-level reflection of spatial-temporal electrophysiological dynamics of the heart of the 12-lead ECG, there are some limitations in its use for ischemic diagnosis, especially in the detection of the ischemic lesion in the heart (Alday et al., 2016).

The newly developed ECG imaging (ECGI) modality provides a promising technology for high-resolution mapping of the electrical and magnetic dynamics of the heart in normal and pathological conditions (Ramanathan et al., 2003; Intini et al., 2005; Rudy, 2017). This approach uses an array of networked electrodes to directly reconstruct the electrophysiological activity of the heart by using the full body surface potential (BSP) and solving the inverse problem in electrocardiography. The inverse problem in electrocardiography is generally ill-posed, this leads to having to use regularisation methods in order to try and constrain the solution (Brooks and Macleod, 1997; Gulrajani, 1998). These regularisation methods have been found to perform differently depending on the heart electrical excitation activity being reconstructed (Figuera et al., 2016; Alday, 2016). Each regularisation method as well as assessing the reconstruction performance has an associated computational cost that requires to be extensively evaluated.

In this work we aim to evaluate the use of ECGI imaging to detect ischemic lesion using a virtual ventricle-torso model. While ECGI may not provide an alternative to conventional imaging techniques (Daly and Kwong, 2013; Carrascosa and Capunay, 2017; Carvalho et al., 2017), it is important to fully evaluate the ECGI technique to assess its strengths and weaknesses and assess how much information could be potentially recovered.

Previous works in to localising ischemia using ECGI has shown the relative performance of two types of Tikhonov regularisation, however the possible effects of using a limited number of electrodes have not been investigated (Messnarz et al., 2004; Ruud et al., 2009). For more clinical relevance in computational ECGI studies, a limited number of recording electrodes should be used, which will have a substantial effect on the overall reconstructed heart surface potential (HSP). As well as HSP's, transmembrane potentials (TMP) have also been explored using a number of methods and frameworks to localise ischemia (Messnarz et al., 2004; Jiang et al., 2009; Wang et al., 2013). A study using a limited number of electrodes developed an optimised 64 electrode layout (Jiang et al., 2009). In this work for simplicity, a grid like structure was used for the each of the electrode layouts (see **Figure 2**).

The focus of this paper is to evaluate the strengths and weaknesses in the performance of the variant regularisation in localising ischemia in combination with different numbers of recording electrodes. Specifically, it aims to evaluate the ECGI ability to reconstruct the ischemic lesion on a ventricle, in order to (i) provide an assessment on the relative performance in regularisation techniques in reconstructing the ischemic lesion; (ii) to compare to how changing the number of electrodes mapping the BSP will affect the accuracy of ischemic lesion reconstruction; (iii) compare the performance of the ECGI varying the size and location of the ischemic lesion; (iv) quantify effects of signal noise on the performance of the regularisation techniques. Further to this, a particular focus has been put on how the error in the detection results manifests itself when using a limited number of electrodes.

This paper is organised as follows. Methods describes the mathematical models used in the computational experiments, the setup and the performance metrics used. Results are split into 3 sections; General Epicardial Potential Reconstructions, Location, Noise and Sensitivity and Lesion Size. These results are then discussed in the following Discussion along with the Limitations and Conclusion.

## Methods

### Forward Problem

The biophysically detailed human ventricular and torso model developed in our previous study (Ni, 2016) was used in this study to simulate the electrical excitation waves in the heart. Full in depth details about the cellular model for the electrical action potentials of the endocardial, midcardial and epicardial ventricular cells, and anatomical structure of the ventricles and torso model can be found in previous studies (Adeniran et al., 2011; Alday et al., 2016; Ni, 2016) which was well validated by the simulated 12-leads ECGs which matched to experimental data. In simulations, the 3D ventricular model was paced at the empirically determined activation sites, mimicking the coupling between the Purkinje fibre network and the ventricles across the endo-surface of ventricular walls (Adeniran et al., 2013; Ni, 2016). The timing of excitation stimulus to the individual activation sites was predetermined, with which the generated ventricular excitation sequence matched to experimental observations and was validated by the simulated 12-lead ECG (Keller et al., 2010; Adeniran et al., 2011; Ni, 2016).

**Figure 1:**
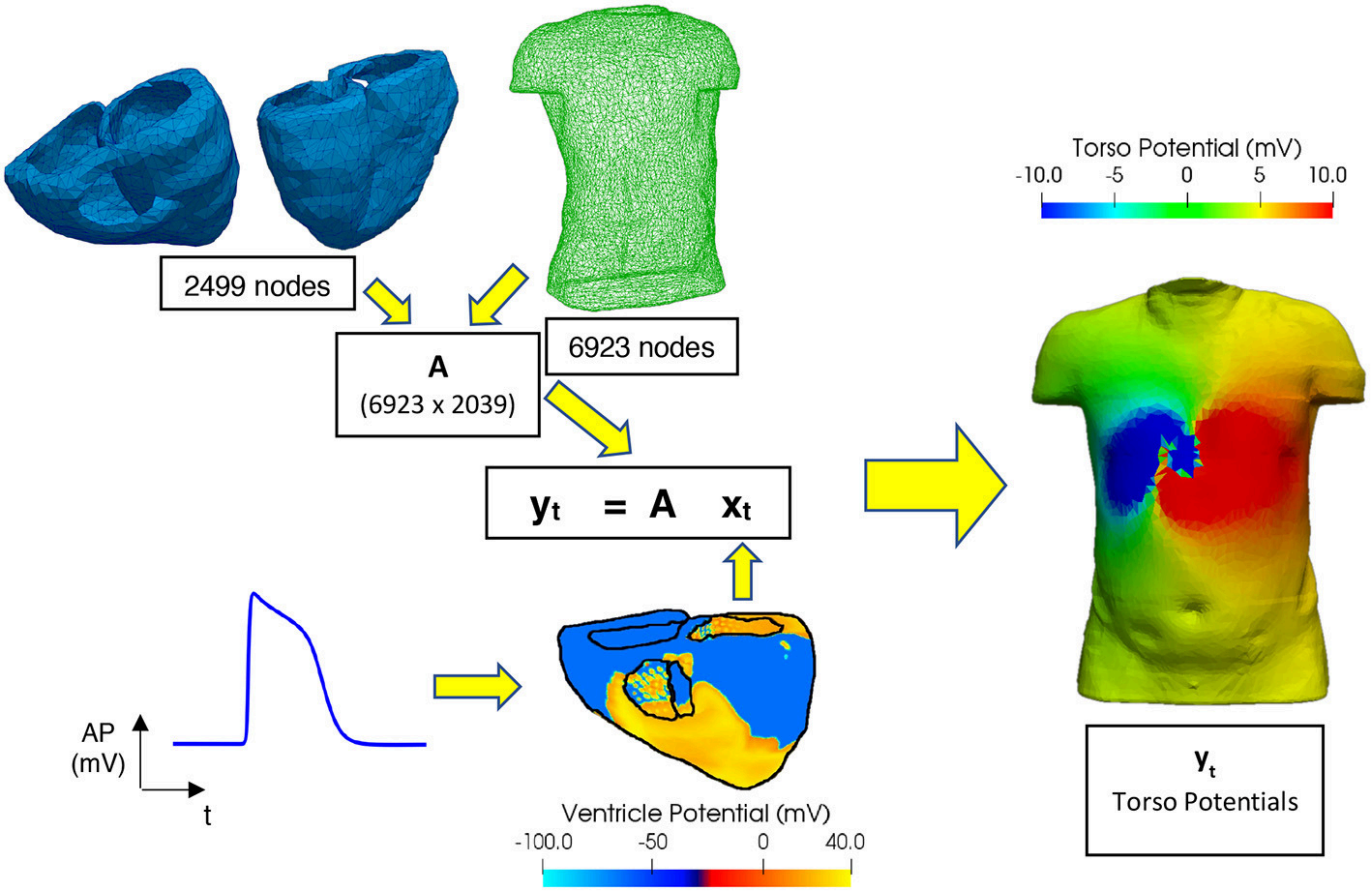
Workflow for performing the forward problem. Transfer matrix **(A)** constructed using a 6,923-node male torso geometry and 2,499-node ventricle geometry, **x**_**t**_ and **y**_**t**_ are matrices filled with the epicardial ventricular potentials and torso potentials at each time t, respectively. Ventricular electrical excitations were simulated using a biophysically accurate 3D computer model of the human ventricles (Ni, 2016). The electrical action potentials on the epicardial surface are extracted and mapped onto the ventricular mesh representing the epicardial surface. Body surface potentials on the torso are then computed using the boundary element method (Stenroos and Haueisen, 2008).

Figure 1 shows the formulation of the forward problem used in this study, including the anatomical geometry of the ventricle and torso meshes used. The ventricle mesh contains 2,499 nodes (5,000 triangular elements) and a male torso mesh contains of 6,923 nodes (13,842 triangular elements), both meshes were down sampled from more detailed geometries used in previous studies (Adeniran et al., 2011, 2013) to reduce the computational time involved in this study. A homogenous torso (isotropic conductivity, i.e., no heterogeneous conductivity introduced by organs such as liver, lungs, ribs, kidneys) is used to create the transfer matrix A in this study considering non-significant effects of these tissues on the ECG (Ramanathan and Rudy, 2001). This also reduces the potential sources of noise to allow comparison of the different regularisation methods. In clinical practice it has been shown that the noise level hides the effect of the torso heterogeneity on the inverse solution (Zemzemi et al., 2015).

In simulations, the ischemic lesion model was idealised as a fully transmural circular zone with a 27 mm radius, unless otherwise stated in the section Location, Noise, and Sensitivity where the size of the ischemic lesion is varied. Similar to previous studies (Jie et al., 2010; Jie and Trayanova, 2011) these lesions consisted of two zones: a central zone and a boundary zone. The central zone consisted of 80% of the radius of the lesion from the centre and the boundary zone the remaining 20%. In the central and boundary zones, cellular electrical remodelling in ion channels and intercellular electrical coupling due to myocardial ischemia were simulated in the same way as in the study of Wang et al. (2013). The parameters associated with the ion channel alteration describing the ischemic zone decrease linearly from central zone to the edge of the boundary zone. As compared to the normal ventricular AP, the ischemic-induced remodelling in ion channel properties resulted in substantial abbreviation in the APD of the ventricular cells (the normal, boundary ischemic zone and central ischemic zone on the right ventricle epicardial myocytes, were 274, 228, and 150 ms, respectively), a markedly depolarised resting potential (by 10 and 18 mV in the border and central ischaemic zone, respectively), and a dramatic reduction in the upstroke velocity of the AP as seen in our previous study (Ni, 2016).

For this study the main lesion placement is on the right ventricle lateral wall (RV LAT). The electrical action potentials on the epicardial surface are extracted and mapped onto the triangulated ventricular mesh representing the epicardial surface. This is done to limit the sources of noise to the sources we can control and quantify and to reduce the computational time. Body surface potentials on the torso are then computed using the boundary element method (Stenroos and Haueisen, 2008) and are used to solve the matrix equation shown in Figure 1.

### Inverse Problem

**Figure 2:**
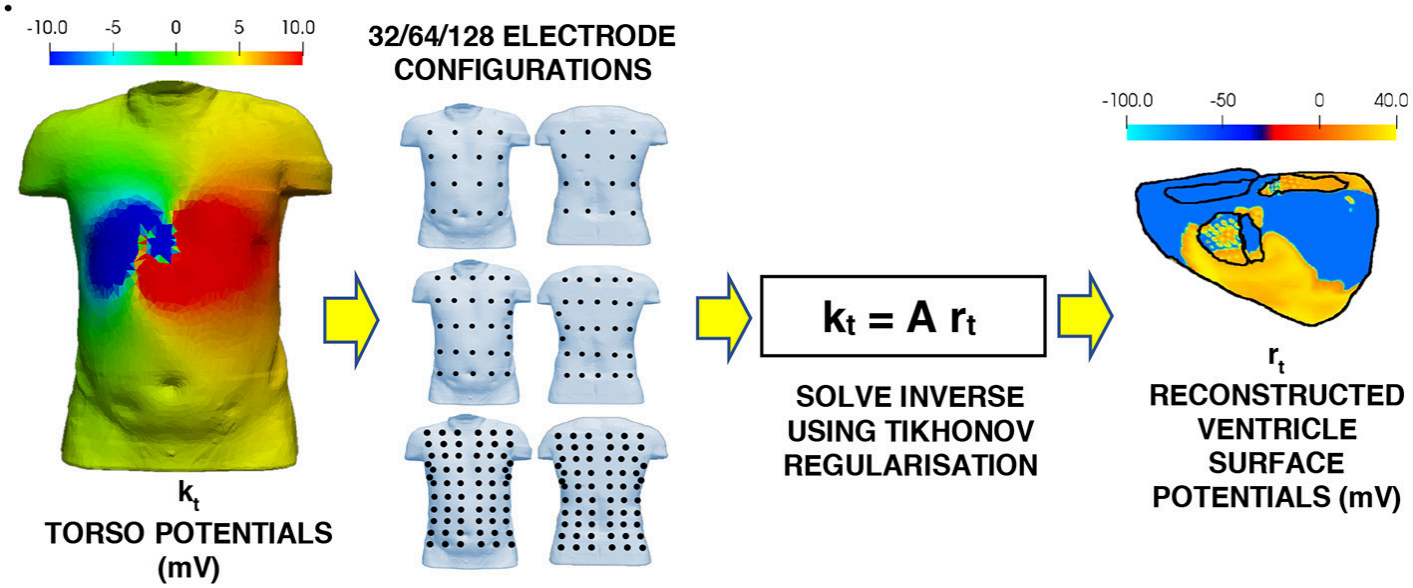
Workflow for the inverse problem. Multi-lead ECG's using the various electrode configurations to the torso potential results from the forward problem. The BSP for the torso is then interpolated from the multi-lead ECG's. This interpolated BSP is then used as the torso potentials in the inverse solution to produce the epicardial potentials on the ventricle.

The complete inverse workflow is shown in Figure 2. The electrode configurations on the torso body surface evaluated in this study are 32, 64, 128, 512, and 1,024 electrodes. The multi-lead ECG's produced by using different electrode configurations, and a linear interpolation algorithm were used to reconstruct the BSP (Oostendorp et al., 1989). The interpolated BSP is then used in the in inverse solution to produce the epicardial potentials from which the ischemic lesion zone can be computed from.

The inverse problem in this study can be represented by the following linear model

(1) $$k_{t}=Ar_{t},$$

where ***k***_*t*_ is the torso potential at time *t* (6,923 × 1 matrix) after reconstruction via interpolation from the torso electrodes, *A* is the transfer matrix formulated entirely from the geometry of the two meshes (Barr et al., 1977) and ***r***_*t*_ is the reconstructed epicardial potential at time *t* (6,923 × 1 matrix). As mentioned previously, this is generally an ill-posed problem which means that simply solving it without any regularisation using an ordinary least squares linear regression will likely lead to a solution dominated by errors. Thus, regularisation techniques are needed to constrain the solution. In this work three regularisation methods were evaluated: 0 order Tikhonov, 1st order Tikhonov and 2nd order Tikhonov.

#### Tikhonov regularisation

The ordinary least squares solution which seeks to minimise the sum of the squared residuals can be written compactly as

(2) $${\left\|Ar_{t}-k_{t}\right\|}_{2}^{2}\;,$$

where $\|\;.{\|}_{2}^{2}$ is the Euclidean norm. In order to penalise undesirable solutions and give preference to desirable solutions a regularisation term is added to the minimisation to form the Tikhonov regularisation (Benning and Burger, 2018).

(3) $${\left\|Ar_{t}-k_{t}\right\|}_{2}^{2}+{\text{λ}}_{t}^{\;2}{\left\|Lr_{t}\right\|}_{2}^{2},$$

Where λ_*t*_ is the regularisation parameter at time *t* and the matrix **L** is the regularisation operator. For each Tikhonov method the Matrix **L** takes a different form

$$\begin{array}{l} \text{zero order }L=\;I, \end{array}$$

first order L= ∇,

$$\text{second order }L=\;\nabla^{2}\;,$$

where **I** is the identity matrix, **∇** is the gradient operator, and **∇**^**2**^ is the Laplacian. Each method favours and penalises the solution in different ways with the gradient operator favouring relatively flat solutions and the laplacian penalising rough solutions in a second derivative sense. The solution to the inverse problem at each time step can then be written as

(4) $$\begin{array}{l} {\hat{r}}_{t}={\left(A^{T}A+{{\lambda_{t}}^{2}L}^{T}L\right)}^{-1}A^{T}k_{t}, \end{array}$$

where ${\hat{r}}_{t}$ is the newly reconstructed epicardial potential. By plotting the log of magnitude term $\left({\left\|Lr_{t}\right\|}_{2\;}^{2}\right)$ vs. the error term $\left({\left\|Ar_{t}-k_{t}\right\|}_{2}^{2}\right)$ yields in most cases an L shaped trade off graph which can be used to identify the most optimal choice for the variable lambda (Hansen, 1992). This optimal λ_*t*_ is found by locating the corner of the L curve, this corner varies in sharpness depending solution and in this study an iterative method (Castellanos et al., 2002; Cultrera and Callegaro, 2016) was used to locate it. In Equation (4), λ_*t*_ would need to be worked out for each time step, requiring an L curve at every time step and thus is quite computationally expensive. In this work the time instants were stacked together into a single matrix **R** and **K**. This allows the computation of just a single λ globally that is independent of time giving the following solution:

(5) $$\begin{array}{l} \hat{R}={\left(A^{T}A+{\lambda^{2}L}^{T}L\right)}^{-1}A^{T}K, \end{array}$$

This approach means that the λ_*t*_ value is not fully optimised for each individual time instant. Testing of using both the single λ_*t*_ for each time instant against the global method for λ determination resulted in the a very small improvement (<0.1) in the average relative difference mean star (RDMS) score (Equation 6) along with a substantial increase in computational time and so the global method was chosen.

### Performance Metrics

Two performance metrics are used in this study, firstly to quantify the overall quality of the epicardial reconstruction and secondly to quantify the quality of the ischemic lesion detection.

#### Relative Difference Mean Star

In this study the relative difference mean star (RDMS) quantifies the amount of error between the reconstructed potentials, $\hat{x},$, and the real potentials, **x**, as in previous inverse studies (Figuera et al., 2016; Gharbalchi et al., 2016):

(6) $$\begin{array}{l} \;RDMS=\;\sqrt{\underset{n}{\sum }{\left(\left(\frac{x_{n}}{\left\|x^{2}\right\|}\right)-\left(\frac{{\hat{x}}_{n}}{\left\|{\hat{x}}^{2}\right\|}\right)\right)}^{2}}. \end{array}$$

This produces a more representative error score then a standard percentage error produces, allowing for better comparison of performance between solutions. There are two ways of calculating this score spatially, in which case you sum over the geometry for each time step, or temporally, in which case you sum over the timesteps for each element in the mesh. Both methods were tested however, the spatial method is the one used throughout this study. This was chosen as it produced more stable error and the average RDMS over all time steps produced a value that represented the data more accurately when visually assessing the epicardial solutions. When comparing RDMS values for different solutions, a lower RDMS value will indicate a better solution.

#### Ischemic Lesion Detection

To detect the ischemic lesion, a threshold approach was implemented in a similar way as previous studies (Wang et al., 2013). Due to the changes in action potential discussed earlier, leads to a lower potential in the central and boundary regions when compared to the surrounding tissue was produced. Thus, if the potential of an element was below a threshold *w*_*threshold*_, then that element was classified as ischemic, else, the element was classified as healthy. To calculate the threshold value the following formula was used

(7) $$\begin{array}{l} \;{\text{w}}_{threshold}=\bar{w}-Q\left(\bar{w}-w_{\min}\right), \end{array}$$

where $\bar{w}$ is the average epicardial potential and *w*_min_ is the minimum epicardial potential and Q is a threshold factor which unless stated otherwise is equal to 0.4. Brief sensitivity analysis on the factor Q is shown in section titled Location, Noise and Sensitivity. Three more metrics can be calculated using this threshold which can help evaluate the performance of the ischemic lesion detection.

- Percentage of ischemic lesion correctly identified as ischemic (CI).
- Percentage of ischemic lesion incorrectly identified as healthy (IH).
- To allow meaningful comparison against (i) and (ii), the area of the ventricle geometry incorrectly identified as ischemic is also displayed as a percentage the total ischemic lesion area (II).

In this study there are 6 variables are considered in total:

- The order of the Tikhonov regularisation (0, 1st 2nd).
- The number of leads used to reconstruct the BSP (32, 64, 128, 256, 512, 1,024).
- The location of the lesion (Right ventricle lateral wall, left ventricle anterior, lateral and posterior wall and interventricular septum).
- The level of gaussian added noise measured as a signal to noise ratio (SNR) in decibels (dB) to the BSP before interpolation (10, 20, 30, and 40 dB).
- The sensitivity of Equation (7), to the factor Q, which will affect the choice of threshold potential used to classify an area as ischemic or healthy (0.3, 0.4, and 0.5 which unless stated otherwise *Q* = 0.4).
- The size of the ischemic lesion (14, 20, and 27 mm).

## Results

### General Epicardial Potential Reconstruction

**Figure 3:**
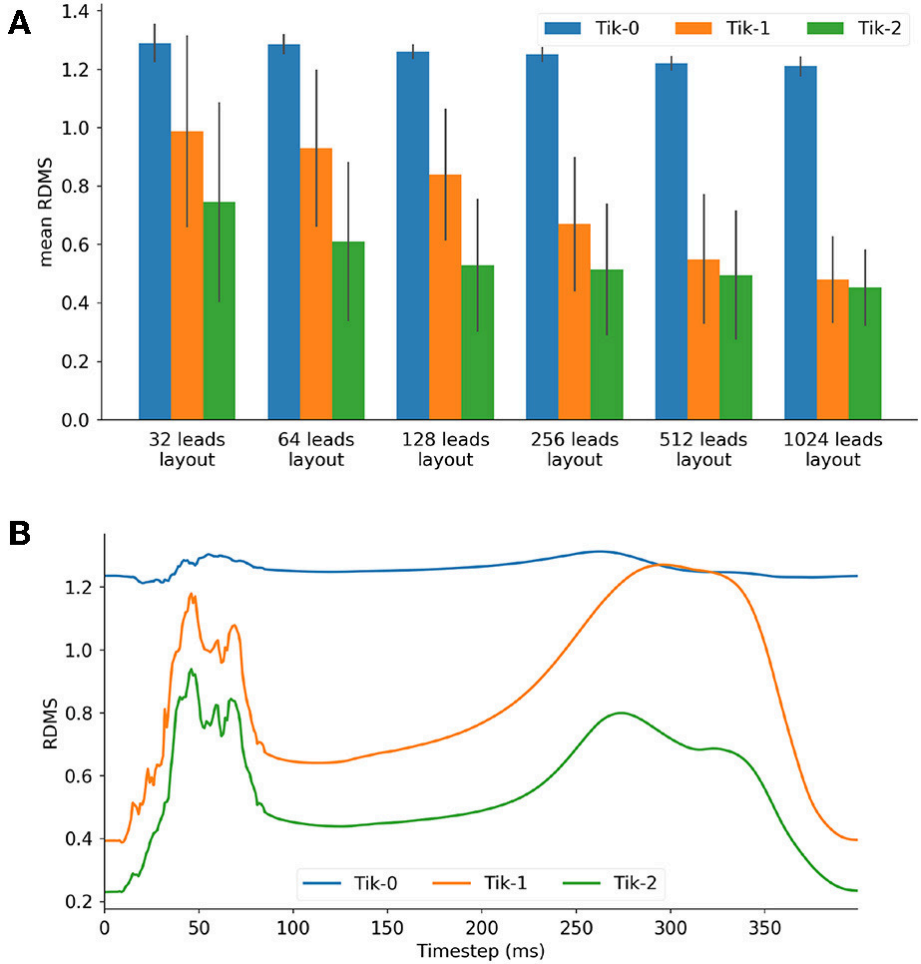
**(A)** Mean RDMS against lead layout, **(B)** 128 lead layout RDMS vs. timestep for the 3 Tikhonov solutions.

Figure 3A shows the mean RDMS (which was calculated spatially and then averaged over all timesteps) for different lead layouts using an increasing number of leads up to 1,024 leads. It can be seen here that all three solutions as expected perform better as the number of leads increases. Both the 1st and 2nd order Tikhonov solutions outperformed the 0 order regardless of how many leads were used. When lead numbers are smaller (less <256) the 2nd order Tikhonov solution clearly outperforms the 1st order solution, however as the lead number increases the difference in mean RDMS between solutions would seem to decrease. At 128 leads the RDMS of the overall 2nd order solution seems to stabilise and not improve greatly as the number of leads increases. Error bars plotted show ±1 standard deviation in the mean RDMS.

**Figure 4:**
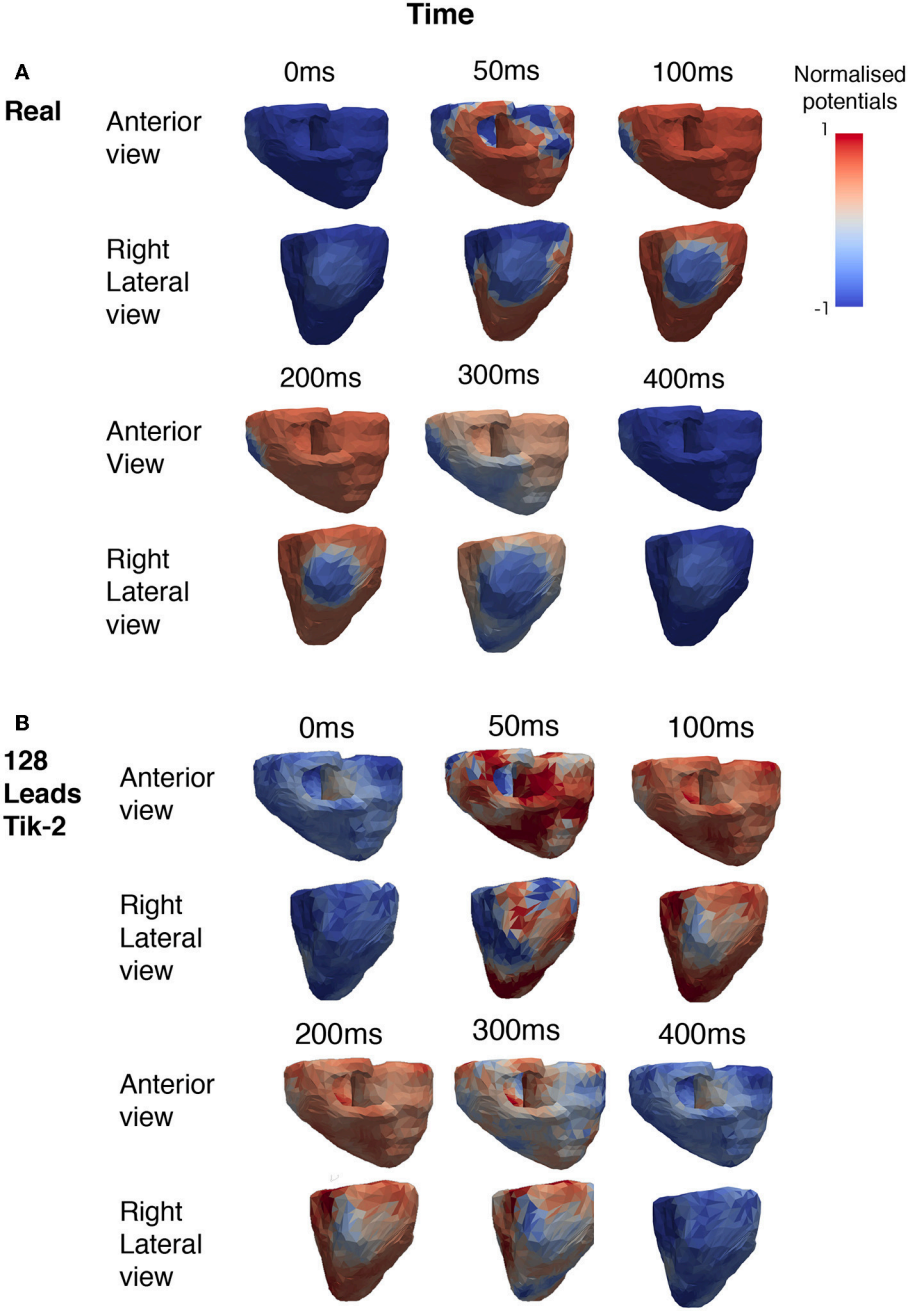
Snapshots showing the comparison between real and reconstructed epicardial excitation pattern. Both anterior and right lateral view of the ventricle were shown for over a period of 400 ms. **(A)** Real epicardial potential patterns at different timings. **(B)** Reconstructed epicardial potential pattern at different timings by using 128 leads and the 2nd order Tikhonov regularisation method.

For the 128 lead solutions the RDMS is plotted against timestep in Figure 3B. The RDMS for the 0 order Tikhonov solution is fairly constant but displays the same characteristics as both the 1st and 2nd order solutions. All solutions show an increasing RDMS as the ventricle depolarises and repolarises while stabilising at a lower RDMS during the plateau phase of the action potentials (AP). These characteristics are present more prominently in the 1st and 2nd order results. If we compare these characteristics with the timesteps shown in Figure 4 there is a correlation between the spatial complexity of the epicardial potential and the RDMS. It can be seen that the 2nd order solution outperforms the 1st order solution which would indicate that the ischemic lesion detection algorithm will perform more accurately.

Figure 4A shows the how the real solution varies over time from two different viewpoints. The first is the anterior view and the second the right lateral view which faces the lesion head on. The full ischemic lesion is clearly visible in the timesteps between 100 and 250 ms, with the maximum range of potential between the ischemic area and the rest of the ventricle occurring at 100 ms. Figure 4B shows the timesteps for the reconstructed solution solved using a 2nd order Tikhonov regularisation. This was chosen as in Figure 3A it was shown to have performed well comparatively and as 128 lead has greater clinical relevance then a larger number (>128) of lead layouts. At 50 ms when the spatial complexity of the real solution is high, we can see how the increased error leads to a pattern that is similar but not very accurate. However, at 100 ms we can clearly make out the presence of an ischemic lesion and general location, but definition is lost in the shape.

### Ischemic Lesion Reconstruction

**Figure 5:**
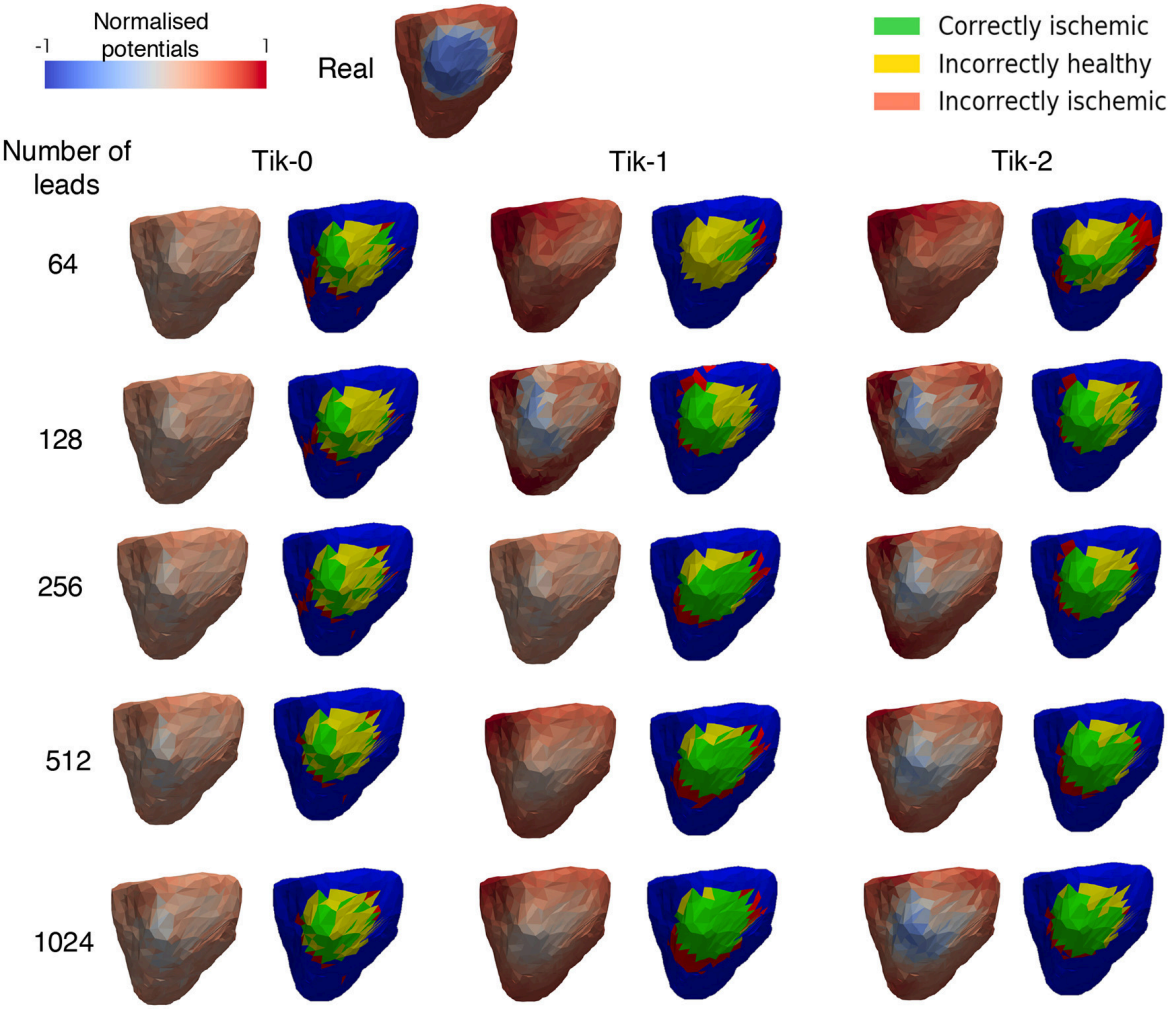
Reconstructed epicardial excitation pattern from the right lateral view of the ventricle at 100 ms. Each regularisation column has two sub columns, **(Left)** is the reconstructed epicardial potential and **(Right)** is a “traffic light” diagram coloured according the ischemia detection results. Green areas represent the successful identification of the ischemic lesion, yellow areas are ischemic which were not identified, and red areas indicate areas incorrectly identified as ischemic.

**Figure 6:**
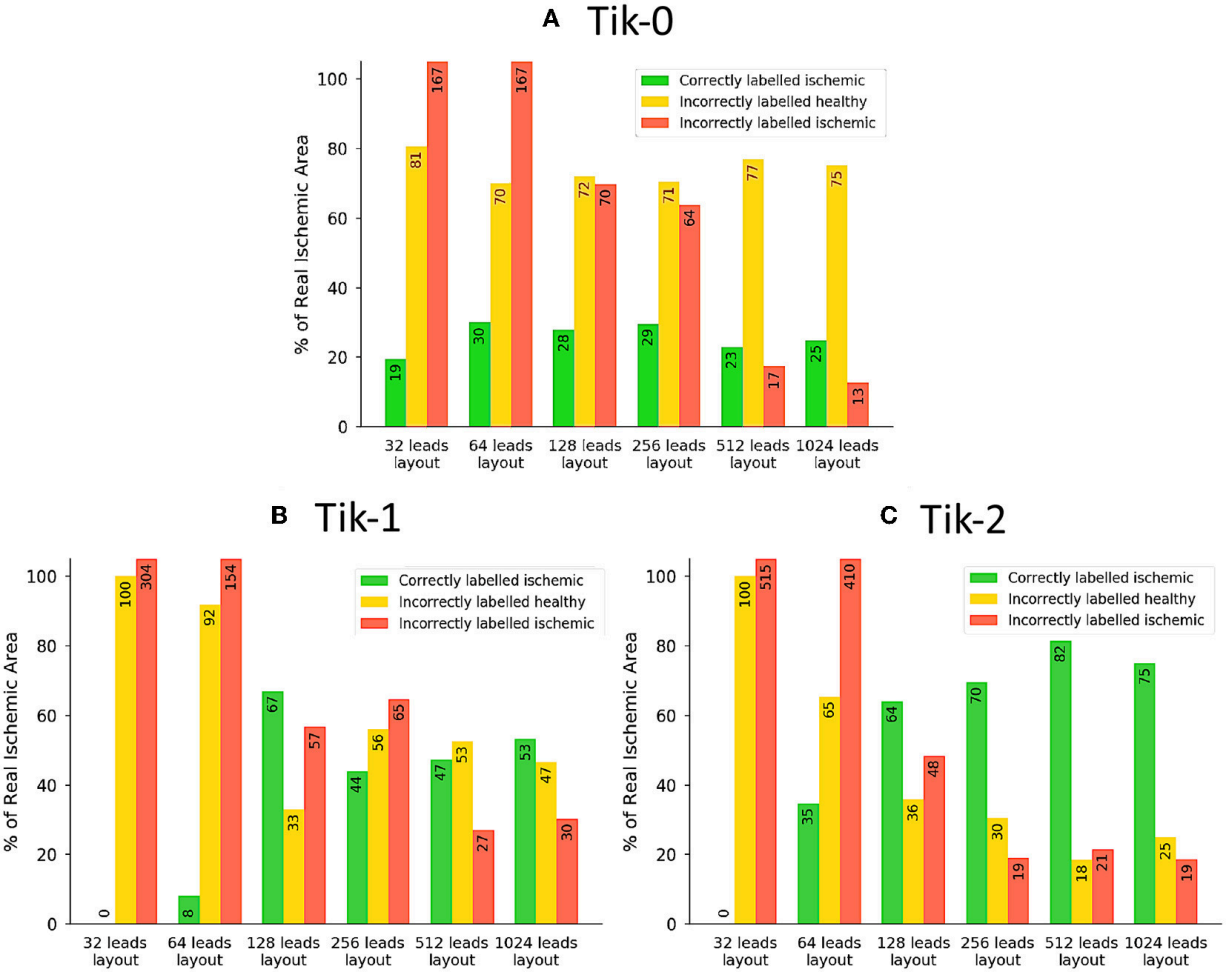
The traffic light data from the ischemic lesion detection visualised as a bar chart. **(A)** 0 order Tikhonov, **(B)** 1st order Tikhonov, and **(C)** 2nd order Tikhonov. Green bars represent the successful identification of the ischemic lesion (correctly ischemic—CI), yellow bars are ischemic which were not identified and shown as healthy (incorrectly healthy—IH) and red bars indicate areas incorrectly identified as ischemic (incorrectly ischemic—II). All area values are represented as a percentage of the real circular lesion. Please note that some bars go above the height of the y axis, bar numbers are present on each bar to aid with this fact.

Figure 5 shows the exemplar results at 100 ms and applying the formula for the threshold ischemic detection described in section Ischemic lesion Detection for all possible combinations of regularisation method and number of leads reconstructing the BSP. This diagram should be viewed in conjunction with Figure 6. It can be seen that the positioning of the correctly identified ischemic lesion in green is generally toward the bottom left of the ischemic lesion. Taking both the green and yellow areas together will show the real ischemic lesion, taking the green and red areas together will show the reconstructed ischemic lesion and taking the red and yellow areas together shows the error in the detection.

**Figure 7:**
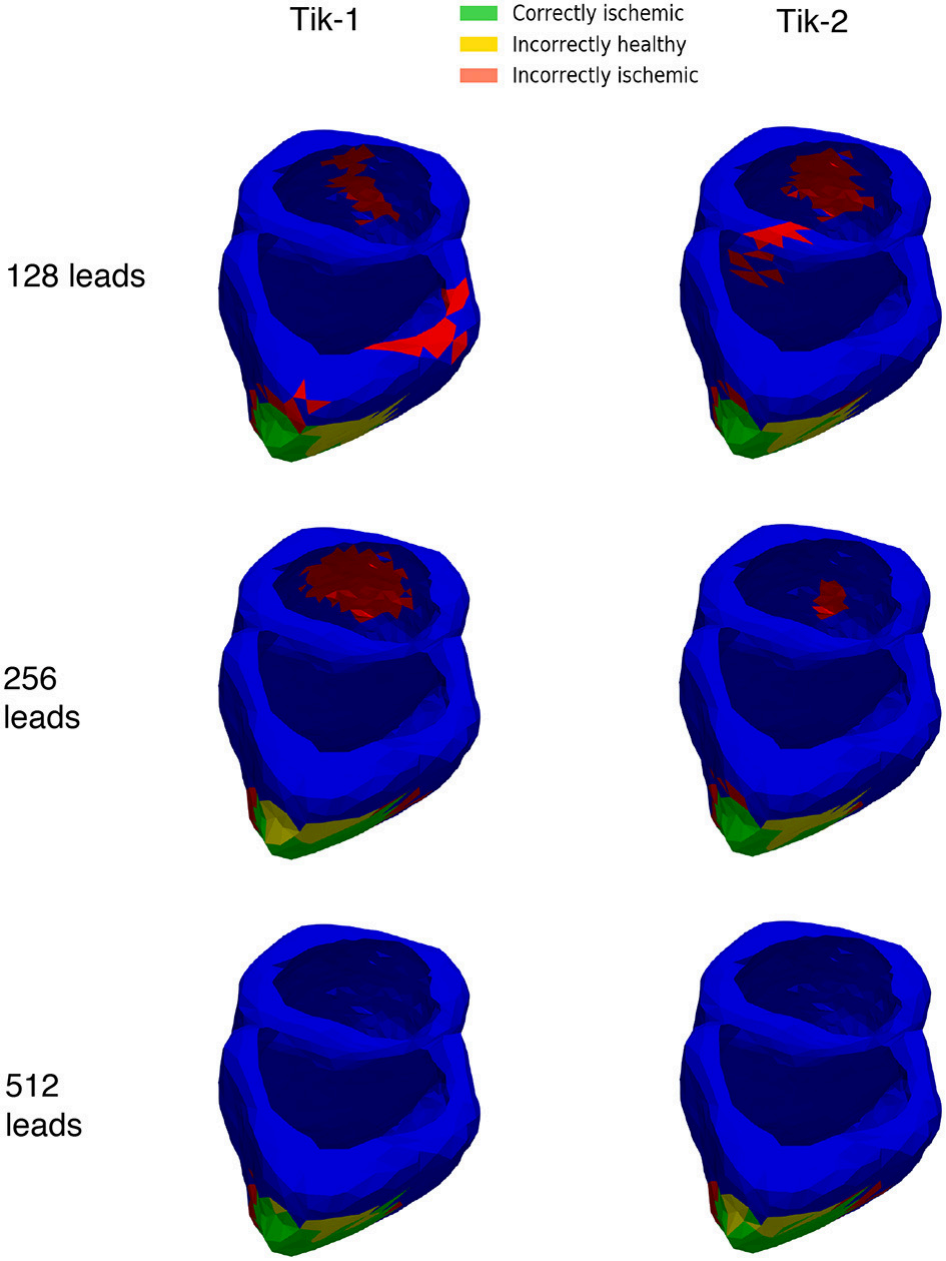
3D ventricle detection results for 1st and 2nd order Tikhonov solutions for 128, 256, and 512 leads. Green areas represent the successful identification of the ischemic lesion, yellow areas are ischemic which were not identified, and red areas indicate areas incorrectly identified as ischemic.

**Figure 8:**
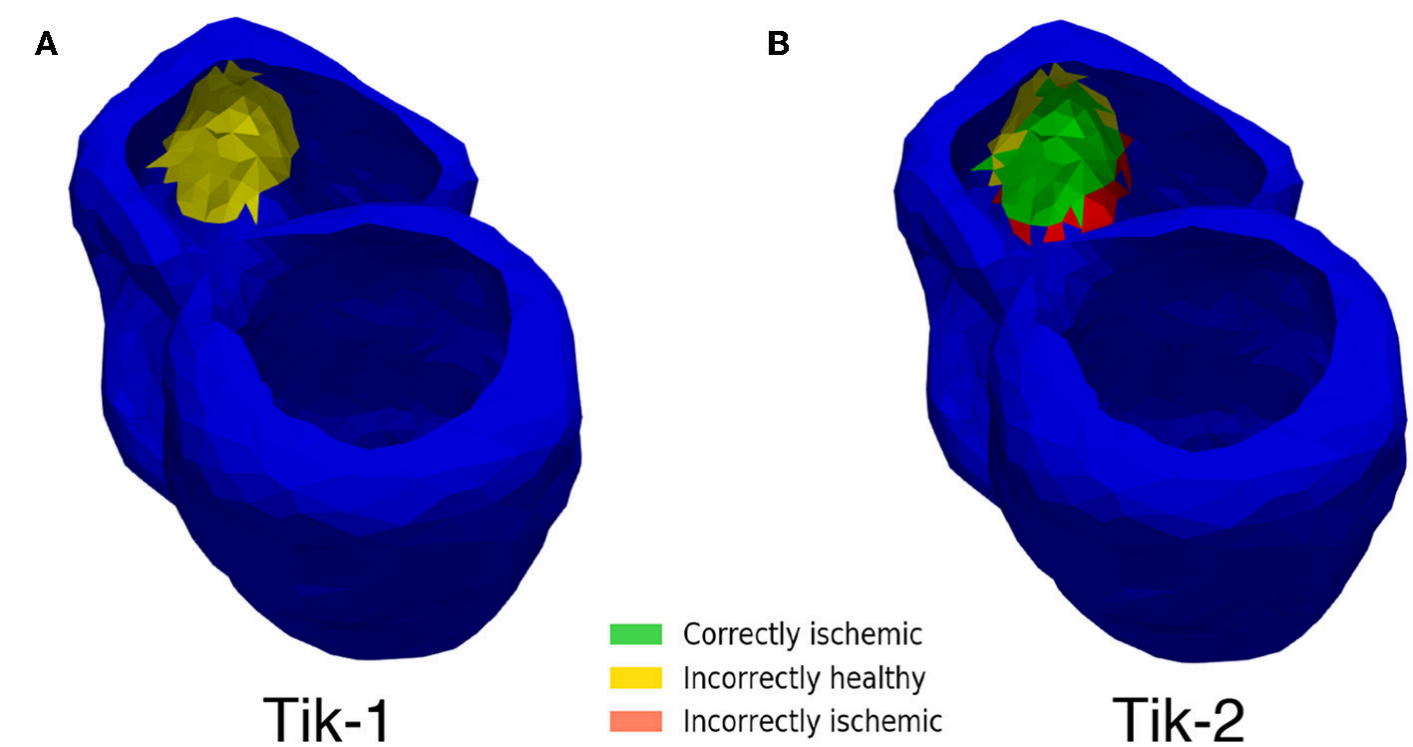
Inner wall lesion detection. **(A)** 1st order Tikhonov 1,024 leads **(B)** 2nd order Tikhonov 1,024 leads.

Much like the results in Figure 3, further confirmation that the increase in leads brings about an increase in the correctly identified ischemic area. It is important to note that the “traffic light” diagrams shown in Figure 5 only shows the right lateral view of the ventricle as that is view most pertinent to this study. However, by looking at the other angles of the ventricle a varying amount of red misidentified ischemic lesions on other parts on the ventricle will be seen depending on the configuration being evaluated. This data is represented in Figures 6–**8** as best as possible, however the full ventricle animations are included in the Supplementary Material.

Using Figures 5, 6A together it can be seen that for the 0 order Tikhonov solution regardless of the number of leads the CI (green) never goes above 30%. However, as number of leads increase the II (red) decreases. The lead layouts from 32 to 256 leads show that even though around 30% of the lesion is detected the II area dominates the overall solution and so even reliably learning the position of the lesion is not possible. The 512 and 1024 lead layouts show much reduced II at 17% and 13%, respectively, this means that by using this configuration it is possible to learn the rough position of the lesion, but nothing about the size and/or shape of the lesion.

Figures 6B,C show at below 128 leads there are not enough leads to accurately map the torso BSP, leading to results that show little CI (green) and very large II (red). Above 128 leads, the results show that increasing the number of leads generally reduces the amount of II. The best ratio of CI:II occur in the 1,024 lead layout solutions for all the regularisation methods. Using Figure 7 it can be seen that as the II decreases as the number of leads increases, the remaining II is more localised around the real lesion. At leads configuration <512 leads it can be seen that a lesion on the inside wall of the left ventricle is erroneously detected in addition to the correct lesion.

Although Figure 5 shows similar results between the 1st and 2nd order Tikhonov solutions in detecting the ischemic lesion, it can be seen that the 2nd order solution outperform the 1st order solutions when considering the full ventricle. This is due the presence of the ischemic lesion on the inside wall of the right ventricle. Figure 8 shows that the 1st order solution struggles to detect any of the inner wall lesion even at 1,024 leads thus leading to a CI of approximately 50%. The 2nd order solution does however perform better in identifying the inner wall lesion, which allows it to reach a CI of around 80% of the total real lesion.

### Location, Noise, and Sensitivity

#### Location

**Figure 9:**
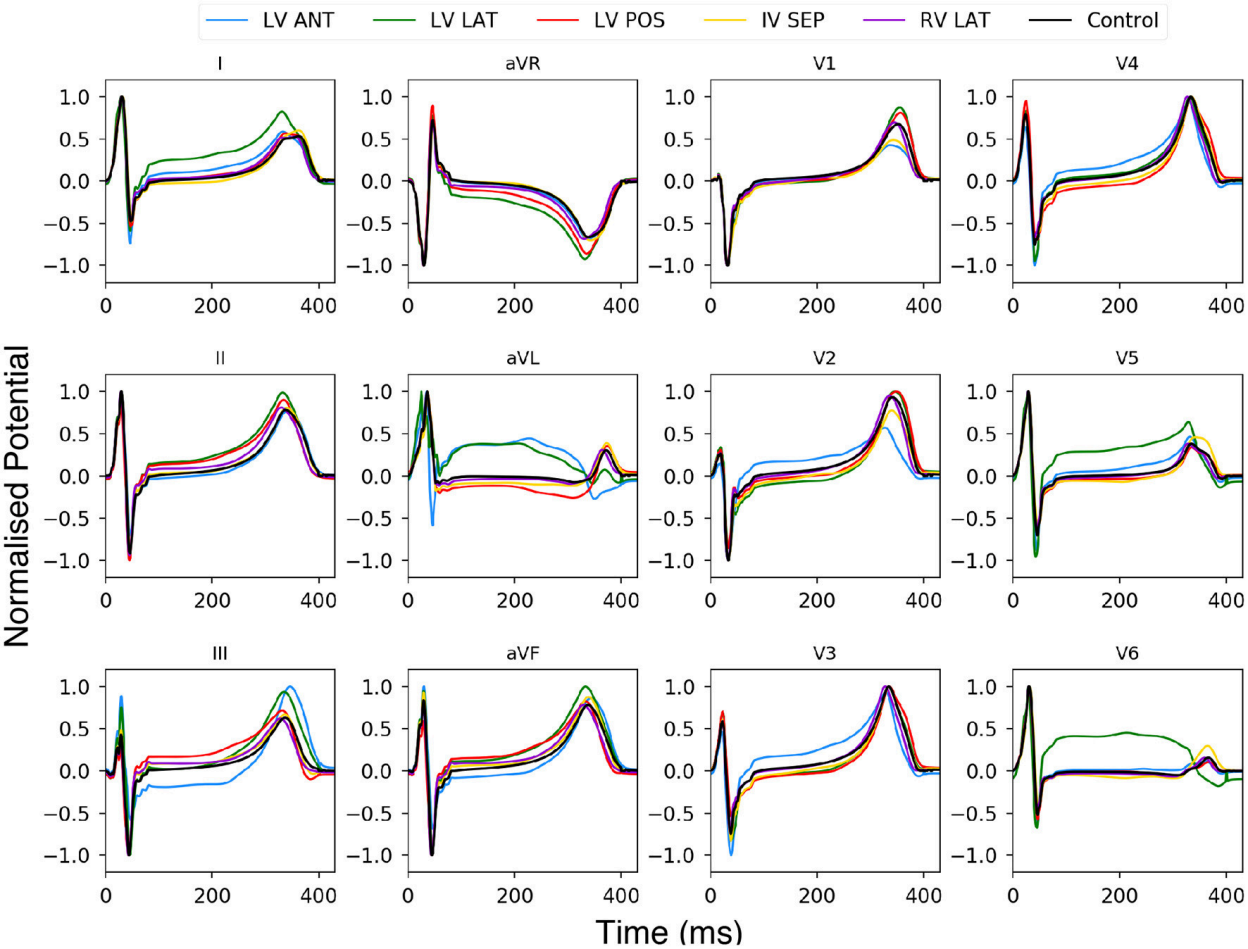
Twelve lead ECG using BSP produced with the human ventricle-torso model. Simulation using healthy control conditions (black) and 5 ischemic lesion locations (5 colours). The y axis has been normalised between 1 and −1.

**Figure 10:**
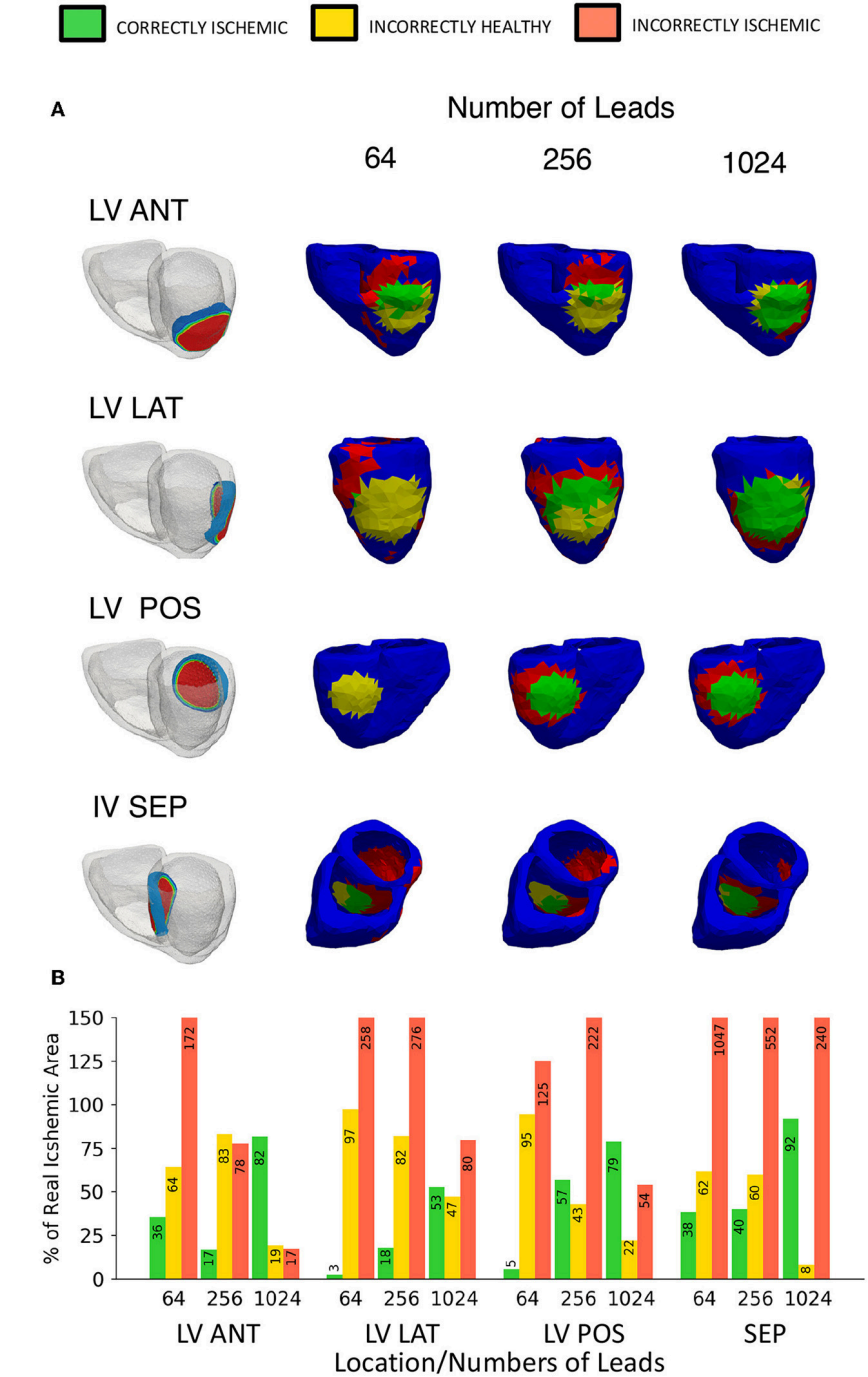
Variable location ischemic lesion detection results using traffic light representation. **(A)** Location vs. number of leads diagram showing the “traffic light” representation of the ischemic lesion detection results, a head on lesion view is shown for each location. First column shows the location of the ischemic lesion on the ventricle, remaining columns show a head on lesion view of the results **(B)** A bar chart visualizing the “traffic light” data taken from the whole geometry as views in **(A)** only show a single view. Please note that some bars go above the height of the y axis, bar numbers are present on each bar to aid with this fact.

In addition to the right ventricle lateral wall location used so far, 4 more locations were also tested. The locations tested in this study are the left ventricle anterior wall (LV ANT), left ventricle lateral wall (LV LAT), left ventricle posterior wall (LV POS), and the interventricular septum (IV SEP). The lesion size was kept constant at 27 mm while only the location and the number of leads were changed. Figure 9 shows the results of 12 lead ECG taken under healthy conditions, and the different ischemic lesion locations. All lesion locations are represented in the first column in Figure 10A, the following columns show the ischemic detection result in each case. As Figure 10A only shows one single view of the three-dimensional ventricle, and due to the limited space available for figures, Figure 10B shows the results of the detection as a bar graph using the data taken from the full geometry.

Using Figure 9 we can see the changes to the ECG profiles for different lesion locations on a 12 lead ECG. The black line is the control case with no lesion present and the coloured lines represent different lesion location over the ventricle. The most prominent change in the profile comes in either an elevated or depressed ST segment, along with both an increase or decrease in the T wave amplitude and then to a lesser degree increases to the amplitudes of the R and S peaks. These changes are consistent with those seen in previous studies (Jie et al., 2010; Hampton, 2013; Perez Alday et al., 2016; Roffi et al., 2016).

Figure 10 shows the equivalent results using the ECGI method, the LV ANT location shows less II error for equivalent number of lead results comparatively with both LV LAT and LV POS. Using the 256 leads LV ANT results we can see that although only 16% was CI, the II error is comparatively low, from the diagram this can be seen as the reconstructed lesion being placed too high on the left ventricle, and thus a high number of leads were then needed to correctly position the lesion further. For the LV LAT and LV POS cases only by using 1,024 leads would lead to successful identification of the lesion position without misidentifying the lesion elsewhere on the ventricle due to error. Similar results were achieved in comparison to the location used with Figures 5, 6 identifying ~80% of the lesion and high lead values. Reconstructions involving the lesion located on the IV SEP performed the worst comparatively having extremely high II error even when using 1,024 leads. Ninety-one percent of the lesion was CI, however this result becomes less useful when viewed along with the II error (240%) which is located on a large percentage of the inside walls of both the left and right ventricle.

Electrograms can be used to indicate and localise ischemia practically through identifying ST elevations. However, whilst ST elevations present in anatomical contiguous leads may strongly suggest ischemia ST elevations are not unique to ischemia and can be caused by a number of other conditions. Localisation of the ischemia can be inferred to regions such as anterior, lateral, apical and septum by observing which leads display the elevations. Using the ECGI technique with more electrodes we are able to learn additional information such as indication of the size, the shape, and location of the lesion.

#### Noise and Sensitivity

**Figure 11:**
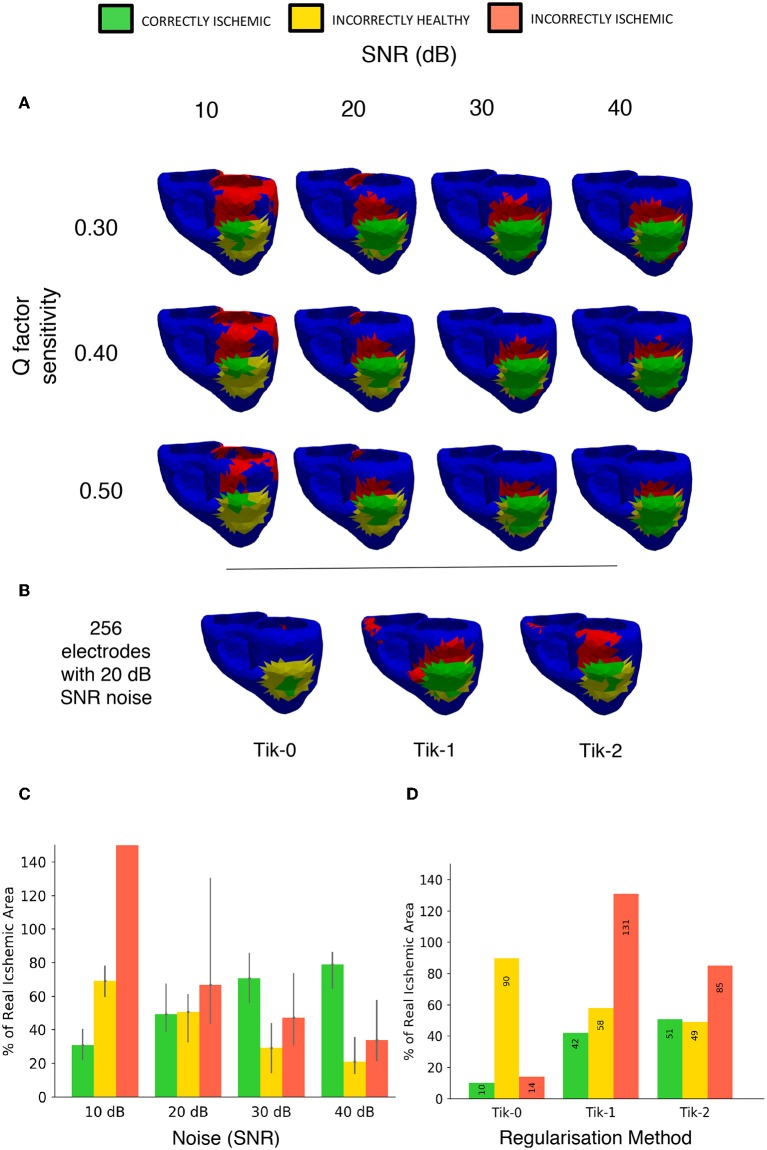
Noise and sensitivity analysis on the lesion detection results and Tikhonov regularisation performance results including 20 dB SNR. **(A)** Signal to Noise ratio (10, 20, 30, 40 dB) vs. lesion threshold algorithm sensitivity with the lesion located on the left ventricle anterior wall. **(B)** Detection results using the 3 different regularisation techniques constructed from BSP's containing noise at 20 dB SNR using 256 electrodes layout. **(C)** Bar chart visualizing the “traffic light” data taken from **(A)** over the whole geometry. Bar height represents data taken from the *Q* = 0.4 in Equation (7) with error bars on the bar showing results of setting *Q* from 0.3 to 0.5. **(D)** Bar chart visualizing the “traffic light” data taken from **(B)** over the whole geometry.

The effects of both noise and our chosen threshold factor Q in Equation (7) were evaluated. The results of 4 different signal to noise ratios measured in decibels and 3 choices for the factor Q are shown in Figure 11A using a lesion on the LV ANT location and 512 leads to interpolate the BSP. As in previous studies, the results show that noise does have a detrimental effect on the performance of the regularised solutions particularly at low SNR (10 dB) leading to low CI and high II. However, over 50% CI is achievable when noise levels reduce as shown by the 20–40 dB columns in Figure 11A. At 10 dB, the II error increase in the case of lesion on the LV ANT location tended to manifest itself increasingly around the top of the left ventricle, thus no real assessment of shape could be made. This same trend occurs to a lesser extent for the detection results using 20–40 dB as the SNR increases.

Figure 11B shows the results with the addition including 20 dB of noise on the relative performance of the 3 regularisation methods using the 256-electrode layout. The order in performance is the same as when not including noise, with the 2nd order Tikhonov performing best, followed by the 1st and 0 orders, respectively. The 2nd order Tikhonov again outperforms the 1st order in terms of identifying the lesion on the inside wall which leads to the two results CI. However, as can be seen in Figure 11B, conversely the 1st order performs better at detecting the lesion on the epicardial wall displaying a more accurate shape and detecting more CI area. At low electrode numbers (<256) with noise included made finding the optimal lambda value using the L curve method more difficult, with the corner of the curve becoming less pronounced and harder to locate. The 2nd order Tikhonov in particular produced L curves which were comparatively more difficult to determine the optimal lambda value compared to the 0 and 1st order Tikhonov solutions.

The Q factor sensitivity is shown in both the rows of Figure 11A and represented as error bars in Figure 11B. As expected using a higher Q factor (0.5) leads to using a lower threshold and thus a smaller area is identified as ischemic, this lead to lower a CI and II in all cases by around 10%. Conversely, using a lower Q factor of 0.3 leads to a higher threshold and thus a larger area is identified as ischemic, this lead to a small increase in the CI however in most cases the II error increased by more thus leading to a poorer overall detection result. The change in Q factor resulted in an either an increased/decreased area detected, this effect was distributed evenly across the both the correct region and the erroneous regions.

### Lesion Size

**Figure 12:**
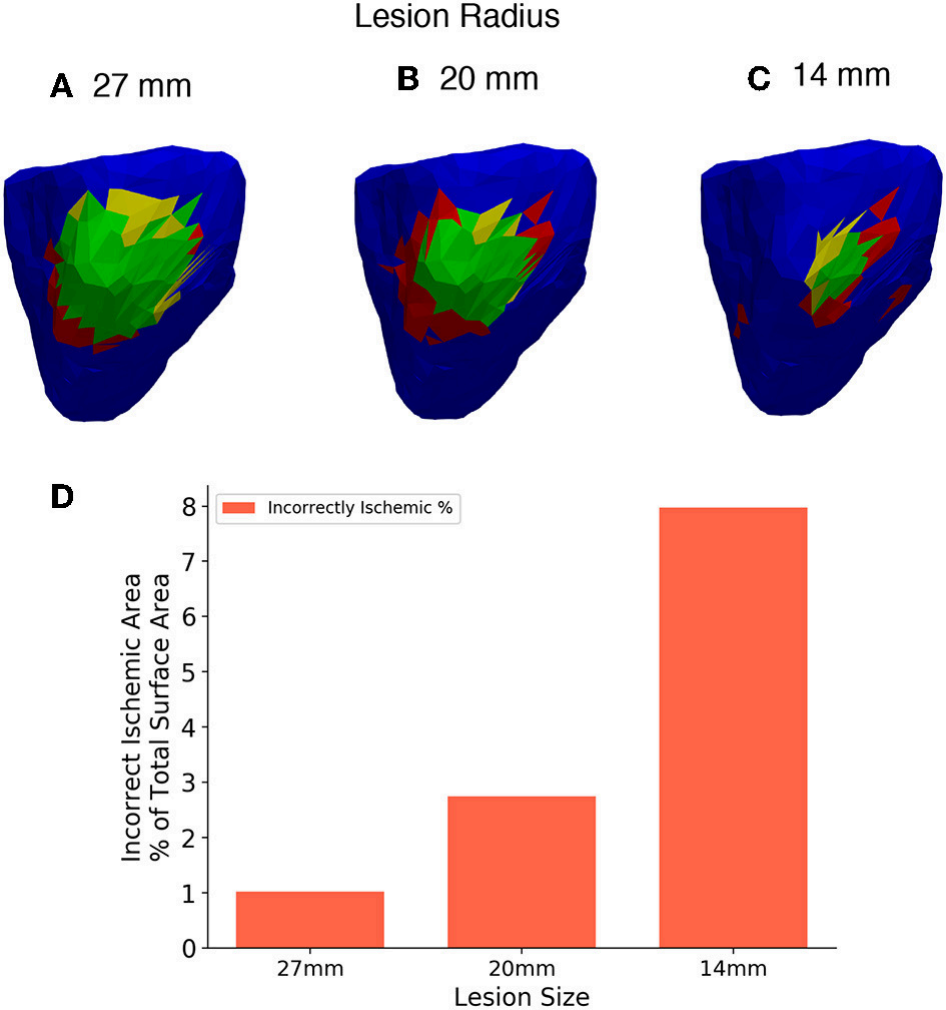
**(A–C)** Three different lesion sizes reconstructed with the 2nd order Tikhonov solution and 512 leads. **(D)** Bar chart showing the areas incorrectly identified as ischemic in each solution as a percentage of the 27mm lesion area for comparison. Right lateral wall view and lesions have radius **(A)** 27 mm, **(B)** 20 mm, **(C)** 14 mm.

Four different sized lesions (labelled A-D) were centred in the same location on the right ventricular lateral wall as in the previous. The radii of the lesions are 27, 20, 14, and 8 mm. Figures 12A–C shows the results of the reconstruction of different sized lesions using the 2nd order Tikhonov regularisation, after the previous sections results showed that most accurate. The 512 lead configuration was chosen to display as this configuration as in the previous analysis it showed the similar results to the 1,024 lead configuration but with fewer leads. The 8 mm radius lesion however could not be reconstructed to any degree even with the highest lead configuration.

The II error increased as the size of the lesion decreased. Thus, there is an increase in the overestimate in ischemic area as the lesion size decreases. Comparison using the charts shown in Figure 6 is not worthwhile in this case as the size of the individual triangular elements of the mesh start to have a large effect on the analysis as the lesion size becomes smaller. However, a limited comparison can made in much the same way as in Figure 6, using only the incorrectly ischemic (II) measurement as shown in Figure 12D. The percentages were calculated in all cases using the 27 mm lesion area as a reference, this allows results between the solutions to be plotted on the same axis. Figure 12D shows the results of incorrectly ischemic error, for all 3 lesion sizes taken from over the whole ventricle geometry. It can be seen that there is an increase in the II error as the lesion size decreases.

## Discussion

In this study, we implemented a ventricle-torso model developed in our previous study to investigate ischemic lesion detection on the ventricle. We quantified the quality of lesion identification while changing the number of leads used to reconstruct the BSP. Effects of 3 different regularisation methods on the lesion detection were also investigated. Smaller investigations into the effect of lesion location and size were also quantified.

### Main Findings

The main findings of this study are that regardless of regularisation technique at least 128 leads are required to reconstruct the ischemic lesion to a level where a location could be established although to reduce the erroneous identification to only localised around the real lesion >256 leads are needed. Using a 0 order Tikhonov solution did not perform well regardless of the number of leads. Using a low number of electrodes (<256), the misidentified ischemic area around the ventricle is large (>70% of the real lesion size), thus this would not produce a solution accurate enough to even identify a rough location for the lesion. At high lead numbers (>256) the misidentified ischemic lesion area reduces in size and are more localised as seen in Figure 7 to the lesion and so a successful accurate location could be identified using these configurations. Further optimisation of the lead positioning could allow for more accurate reconstructions at lower leads numbers (Jiang et al., 2009).

Using the 1st order Tikhonov solution gives a good solution for the outer lesion area, even outperforming the 2nd order, but fails to identify the lesion on the inner wall, thus leading to solutions which at maximum identify about 53% of the total lesion area. With increasing number of leads the solution produce less error, and the error becomes more localised around the lesion in contrast to lower leads solutions. The 2nd order Tikhonov solution proved to be the best of the three regularisation methods, both in terms of error and amount of correctly identified ischemic lesion. It also outperformed the 1st order solution identifying more successfully the inner wall ischemic lesion leading to around 80% lesion area identification. This agrees with previous work to localise ischemia which show results in which the 2nd order Tikhonov performs better (Messnarz et al., 2004; Ruud et al., 2009) and this still stands when using a limited number of recording electrodes with no noise.

Varying the location of the lesion showed that ischemic lesion detection performs more accurately for LV ANT location and LV POS then LV LAT, with solutions achieving lower comparative II error using the same number of leads. This follows along with what was expected as the distance between the ventricle and torso is smaller here along with being more aligned with the layout of the leads which are heavily skewed mainly towards the front and back of the torso. Finally, the lesion located on the IV SEP showed comparatively less accurate results, this is expected as the outer walls of the ventricle shield and mask the effect of the lesion on the BSP to a certain degree. Although results indicated that a lesion on the inner walls of the geometry was present, it would not be accurate enough to successfully identify the lesion size and location. A more accurate BSP measurement is needed to improve the solution to a successful level, requiring either more leads or a more accurate interpolation method.

The addition of noise causes the solutions to reduce in detection accuracy. The 2nd order solution proves to be more accurate when looking at both the inner and outer walls of the ventricle, however if the criteria was changed to only recognise the outer epicardial layer the 1st order solution proved to be better. The addition of noise caused noticeable change in the sharpness of the L curve used to optimise the selection of the regularisation parameter lambda in combination with a limited number of electrodes. The 2nd order solution in particular was most affected by this. As the choice of lambda value has such a pivotal role in the ECGI workflow, determining the optimal value needs to be accurate and clear, perhaps another method (Golub et al., 1979; Pei et al., 2015; Barnes and Johnston, 2016; Chung and Español, 2017) would prove to be more useful in high noise low recording electrode configurations. Taking ST-integral of the BSP electrograms instead of just one timestep as used in this study could also help reduce the effect of measurement noise as employed in a previous study (Jiang et al., 2009).

As expected the smaller the lesion size the less accurate the lesion reconstruction becomes. Using this current setup accurate lesion detection was achieved at a lesion size of 20 mm and above using 512 leads. Decreasing lesion size also caused an increase in the overestimate of the lesion size when reconstructed. However, the 14 mm lesion was detected but along with a substantial area of incorrect regions that should have been detected as healthy areas of the ventricle as seen in Figure 12C. Therefore, this setup is not well suited for detecting lesions of that size or smaller. As a benchmark the 14 mm lesion was reconstructed without the use of recording electrodes and thus no interpolation and similar results were achieved but with slightly less error (~6% using the same scale as Figure 12D) and so even with a perfect BSP reconstruction 14 mm lesions cannot be detected accurately using this workflow. Further study is needed to see if using a more detailed mesh (>5,000 triangles) would allow for a more accurate reconstruction of the smaller lesion. Using a more detailed mesh increases the complexity of the problem, as well as its “ill-posedness” and so more accurate results are not guaranteed.

## Limitations

There are a number of limitations with this study which should be considered when interpreting the results. Firstly, a global method was used to produce a single lambda value for the regularisation across all timesteps to generate the RDMS values shown in Figure 4. An individual timestep approach was implemented but the results provided only a marginal improvement whilst also requiring substantially more computing time. There are other regularisation methods (Figuera et al., 2016; Benning and Burger, 2018) which may or may not improve the solution however these have not been tested in this study. The shape of the lesion is idealised to be circular for computational/modelling simplicity, it is not yet clear how more complex/multiple lesions will perform. The smoothing properties of inverse regularisation methods may impact these complex shapes differently and will have to be further investigated. This study has not been tested and validated in a clinical setting, however computer modelling provides a direct comparison between results computed from solving the forward and inverse problem which can then be used as a basis for clinical studies. Finally, clinically electroanatomic mapping (EAM) studies have shown that using general unipolar electrogram thresholds can be unreliable in detecting scar, as unipolar voltages show large variations even in non-scar locations and are effected by tissue fat (Nguyên et al., 2017). Bipolar electrograms have been shown to be more reliable (Codreanu et al., 2008) and so further study is warranted to test and evaluate the use of different metrics to evaluate ECGI reconstructions.

## Conclusion

A ventricle-torso model was used to investigate the use of ECGI as a tool to detect ischemic lesions. We quantified the quality of lesion identification while changing the number of leads used to reconstruct the BSP. The results show that the 2nd order Tikhonov is the best of the three regularisation methods tested in this study, showing and detecting a circular ischemic lesion of 27 mm with lead numbers >256. At >256 leads the 2nd order Tikhonov showed not only a higher % of lesion detected but a reduced and more localised error around the lesion itself. Further to this, the 2nd order Tikhonov proved to be more accurate identifying the lesion on the inner wall of the ventricle. This superior inner wall detection still proved to be the case when noise was added into the workflow, however the relative performance gap between then 1st and 2nd order Tikhonov is reduced. The combination of both noise and a limited number of recording electrodes produced “L curves” with a less pronounced corner when viewed comparatively against their no noise counterparts. Lesions on the left ventricle walls were also able to be identified but comparatively to the right ventricle lateral wall performed marginally worse, with lesions located on the interventricular septum being able to be indicated by the reconstructions but not successfully identified against the error.

## Author Contributions

VK and HZ conceived the experiments. VK performed the code implementation, development, testing, and analysis. HN and EP contributed computing resources. VK, HN, and HZ contributed to the manuscript.

### Conflict of Interest Statement

The authors declare that the research was conducted in the absence of any commercial or financial relationships that could be construed as a potential conflict of interest.

## Supplementary Material

Video S1 Ventricle reconstruction using 0 order Tikhonov and 1024 leads.

Video S2 Ventricle reconstruction using 1 order Tikhonov and 1024 leads.

Video S3 Ventricle reconstruction using 2 order Tikhonov and 1024 leads.

Video S4 Ventricle detection result using 0 order and 1024 leads.

Video S5 Ventricle detection result using 1 order and 128 leads.

Video S6 Ventricle detection result using 1 order and 1024 leads.

Video S7 Ventricle detection result using 2 order and 128 leads.

Video S8 Ventricle detection result using 2 order and 1024 leads.
