# Corrigendum: ECG Imaging to Detect the Site of Ventricular Ischemia Using Torso Electrodes: A Computational Study

**DOI:** 10.3389/fphys.2019.00692

**Published:** 2019-05-29

**Authors:** Vinay Kara, Haibo Ni, Erick Andres Perez Alday, Henggui Zhang

**Affiliations:** ^1^Biological Physics Group, School of Physics and Astronomy, The University of Manchester, Manchester, United Kingdom; ^2^Department of Pharmacology, The University of California, Davis, Davis, CA, United States; ^3^Division of Cardiovascular Medicine, Oregon Health and Science University, Portland, OR, United States; ^4^School of Computer Science and Technology, Harbin Institute of Technology, Harbin, China; ^5^China Space Institute of Southern China, Shenzhen, China

**Keywords:** ECGI, ventricle, torso electrodes, inverse problem, ventricular ischemia, regularisation methods

There is an error in the **Funding** statement. The correct number for “EPSRC” is “(EP/J00958X/1; EP/I029826/1).”

The authors apologize for this error and state that this does not change the scientific conclusions of the article in any way. The original article has been updated.

